# Quantification of *Lyssavirus*-Neutralizing Antibodies Using Vesicular Stomatitis Virus Pseudotype Particles

**DOI:** 10.3390/v8090254

**Published:** 2016-09-16

**Authors:** Sarah Moeschler, Samira Locher, Karl-Klaus Conzelmann, Beate Krämer, Gert Zimmer

**Affiliations:** 1 Institut für Virologie und Immunologie (IVI), Abteilung Virologie, CH-3147 Mittelhäusern, Switzerland; sarah.moeschler@ivi.admin.ch (S.M.); samira.locher@ivi.admin.ch (S.L.); 2 Max von Pettenkofer-Institut und Genzentrum, Ludwig-Maximilians-Universität, D-81377 München, Germany; conzelmann@lmb.uni-muenchen.de; 3 Paul-Ehrlich-Institut, Abteilung Veterinärmedizin, D-63225 Langen, Germany; beate.kraemer@pei.de

**Keywords:** virus neutralization, pseudotype virus, rabies virus, luciferase, biosafety, vaccine, immunoglobulin

## Abstract

Rabies is a highly fatal zoonotic disease which is primarily caused by rabies virus (RABV) although other members of the genus *Lyssavirus* can cause rabies as well. As yet, 14 serologically and genetically diverse lyssaviruses have been identified, mostly in bats. To assess the quality of rabies vaccines and immunoglobulin preparations, virus neutralization tests with live RABV are performed in accordance with enhanced biosafety standards. In the present work, a novel neutralization test is presented which takes advantage of a modified vesicular stomatitis virus (VSV) from which the glycoprotein G gene has been deleted and replaced by reporter genes. This single-cycle virus was *trans*-complemented with RABV envelope glycoprotein. Neutralization of this pseudotype virus with RABV reference serum or immune sera from vaccinated mice showed a strong correlation with the rapid fluorescent focus inhibition test (RFFIT). Importantly, pseudotype viruses containing the envelope glycoproteins of other lyssaviruses were neutralized by reference serum to a significantly lesser extent or were not neutralized at all. Taken together, a pseudotype virus system has been successfully developed which allows the safe, fast, and sensitive detection of neutralizing antibodies directed against different lyssaviruses.

## 1. Introduction

Rabies is a fatal neurological disorder in humans and other mammalian species which is caused by rabies virus (RABV), the prototype member of the genus *Lyssavirus* within the family *Rhabdoviridae* [1]. The *Lyssavirus* genus currently contains 14 classified and one proposed species which are classified into distinct phylogenetic groups [2,3,4,5,6,7,8]. Group 1 consists of RABV, Khujand virus (KHUV), Australian bat lyssavirus (ABLV), European bat lyssavirus type 1 (EBLV-1), European bat lyssavirus type 2 (EBLV-2), Bokeloh bat lyssavirus (BBLV), Aravan virus (ARAV), Duvenhage virus (DUVV), and Irkut virus (IRKV). Shimoni bat virus (SHIBV), Lagos bat virus (LBV), as well as Mokola virus (MOKV) represent group 2, while West Caucasian bat virus (WCBV) represents group 3 [5,6]. For Ikoma and Lleida bat lyssaviruses (IKOV and LLEBV, respectively) the establishment of a novel group 4 has been proposed [7,8].

The prototype RABV has a worldwide distribution and is found primarily in carnivores (e.g., dogs, foxes, raccoons, skunks, wolves, etc.) and North American (but not European) bats. Except MOKV, which has been found in shrews and cats, all other lyssaviruses have their natural reservoir in bats. By far, most human cases of rabies are caused by RABV, but sporadic infection with other *Lyssavirus* species has been reported to cause the disease as well [9,10,11,12].

RABV is usually transmitted to humans through saliva following a bite from an infected animal. The virus migrates to the central nervous system (CNS) via axonal retrograde transport and trans-synaptic transmission. Depending on the site of inoculation and the viral load in the inoculum this can take several weeks during which symptoms are not apparent. Usually, anti-RABV antibodies cannot be detected during this incubation period, thus impeding early serological diagnosis. Once the virus has reached the CNS, the outcome of the infection is almost always fatal [13].

Inactivated rabies vaccines have been approved for immunoprophylaxis of animals as well as humans who are at risk of exposure to RABV (e.g., veterinarians, laboratory workers). The vaccine provides protection by inducing virus-neutralizing antibodies directed to the single viral envelope glycoprotein G [14]. Thanks to the obligatory vaccination of pet dogs and cats and campaigns for vaccination of wildlife, some European countries are now declared rabies-free. The RABV vaccine protects against infection with members of phylogroup 1, but not of phylogroup 2 [15].

A post-exposure prophylaxis is available as well and includes administration of immunoglobulins from vaccinated humans or horses and accompanying active immunization with the inactivated RABV vaccine. This therapy is effective in preventing rabies disease only if given immediately after exposure to the virus. About 15 million people receive this post-exposure treatment every year following potential exposure to RABV. Despite being preventable by pre- and post-exposure prophylaxis, RABV still causes about 50,000 human deaths per year, mostly in India, China, and African countries [16]. This is mainly due to the low availability and accessibility of vaccines and immunoglobulin therapy in these countries, but also because RABV is not effectively controlled in stray dogs [17].

In order to assess the quality of immunoglobulin preparations, as well as the immune status of vaccinated animals and humans, a RABV neutralization test is usually performed. The fluorescent antibody virus neutralization (FAVN) test and the rapid fluorescent focus inhibition test (RFFIT) are the currently approved methods for the quantification of neutralizing antibodies [18,19]. Both require handling of live virus, making use of appropriate biosafety containment as well as vaccination of laboratory personnel necessary [20]. Recently, an alternative virus neutralization assay has been developed which is based on lentiviral pseudotypes [21].

Vesicular stomatitis virus (VSV), like RABV, is a member of the family *Rhabdoviridae*, but has been classified into the genus *Vesiculovirus*. In the present work, a glycoprotein-G-deficient VSV expressing two different reporter genes was pseudotyped with lyssavirus envelope proteins using stably transfected helper cell lines. The VSV pseudotype particles and reporter gene expression were employed for virus neutralization tests using immune sera from vaccinated animals, World Health Organization (WHO) reference serum, and neutralizing antibodies. Specificity, sensitivity, and time requirements of this new pseudotype neutralization test were determined and compared with those of a commonly used RFFIT test.

## 2. Materials and Methods

### 2.1. Cells

Vero cells (C1008) were purchased from the American Type Culture Collection (Manassas, VA, USA) and maintained in Glasgow’s minimal essential medium (GMEM; Life Technologies, Zug, Switzerland) supplemented with 5% fetal bovine serum (FBS). Baby Hamster Kidney (BHK)-G43, a transgenic BHK-21 cell clone expressing the VSV G protein in a regulated manner [22], was maintained in minimal essential medium (MEM) containing 5% FBS. T-Rex™-Chinese Hamster Ovary (CHO) cells were purchased from Life Technologies and maintained in Ham’s F12 medium (Sigma-Aldrich, Buchs, Switzerland) supplemented with 5% FBS.

### 2.2. Generation of Helper Cell Lines

For the generation of transgenic helper cell lines, cDNA encoding the following viral envelope proteins was used: VSV (serotype Indiana) G protein (GenBank accession number: J02428), rabies challenge virus standard 11 (CVS-11) G protein (GenBank accession number: EU126641) [23], RABV vaccine strain Street-Alabama-Dufferin B19 (SAD B19) G protein (GenBank accession number: M31046) [24], European bat lyssavirus 1 (EBLV-1) G protein (GenBank accession number: EF157976) [25], and Mokola virus (MOKV, strain Ethiopia 16) G protein (GenBank accession number: U17064) [26]. A codon-optimized cDNA encoding the G protein of European bat lyssavirus 2 (EBLV-2, isolate RV1787) (GenBank accession number: EU352769) was purchased from GenScript (Piscataway, NJ, USA). The cDNAs were amplified by PCR and inserted into the pcDNA™ 4/TO/Myc-His plasmid (Life Technologies) downstream of the cytomegalovirus (CMV) promoter and tetracycline operator sites. T-Rex™-CHO cells were transfected with recombinant plasmid and Lipofectamine 2000 (Life Technologies). The cells were grown for two weeks with medium containing 1 mg/mL zeocin (Invivogen, Toulouse, France) and 5 µg/mL blasticidin (Invivogen). Surviving cells were subsequently cloned by limiting dilution.

### 2.3. Antibodies and Immune Sera

A monoclonal antibody directed to the RABV G protein (hybridoma clone 16DB4) was provided by the Swiss Rabies Centre (Bern, Switzerland). The second international standard for human anti-RABV immunoglobulin (reference serum) was obtained from the National Institute for Biological Standards and Control (NIBSC, Potters Bar, U.K.), and reconstituted in water to give a final concentration of 2 IU/mL.

Serum samples were obtained from mice (NMRI strain), which had been vaccinated twice (one week apart) with 0.5 mL of WHO standard RABV vaccine (Sixth International Standard for Rabies vaccine, 8 IU/mL, NIBSC). Three different vaccine doses (1/10; 1/50; 1/250) were used and applied to the animals (group size *n* = 10) via the intraperitoneal route. Two weeks after the second immunization the animals were bled and sera prepared. The mouse immunization experiments were approved by the regional council in Darmstadt (authorization number V54-19c20/15-F107/104) and performed at the Paul-Ehrlich-Institute in Langen, Germany, in compliance with German animal protection law.

For generation of polyclonal antibodies directed against the G proteins of MOKV and CVS-11, recombinant vector vaccines based on propagation-incompetent VSV were produced [27]. The ectodomains of the glycoproteins (amino acids 1–439 for CVS-11 G and 1–448 for MOKV G) were genetically fused to the GCN4_pII trimerization domain and inserted into the fourth transcription unit of the pVSV*ΔG(HA) plasmid [27] resulting in pVSV*ΔG(secMOKV-G) and pVSV*ΔG(secCVS-G). The recombinant viruses were generated and propagated in BHK-G43 helper cells as described previously [28]. Two rabbits were immunized intramuscularly (i.m.) with 10^8^ focus-forming units (f.f.u.) of either VSV*ΔG(secMOKV-G) or VSV*ΔG(secCVS-G) in the absence of adjuvant. The immune response of the animals was boosted using the same vaccine four weeks after the primary immunization. After four more weeks, the animals were boosted a second time by intramuscular injection of 20 µg of pCAGGS plasmid DNA encoding the G proteins of CVS-11 and MOKV, respectively. The DNA was formulated with 20 µL of Lipofectamine® 2000 (Life Technologies). The animals were euthanized and bled four weeks after the final immunization. These animal experiments were performed in compliance with the Swiss animal protection law and approved by the animal welfare committee of the canton of Bern (authorization number BE119/13).

### 2.4. Immunofluorescence Analysis

Transgenic T-Rex™-CHO cell lines were cultured for 24 h on 12 mm glass coverslips with Ham’s F12 medium containing 5% FBS and 2 µg/mL of doxycycline (Sigma-Aldrich). Control cells received medium without doxycycline. The cells were washed with phosphate-buffered saline (PBS) and incubated with primary antibodies for 60 min on ice. For detection of the G proteins of CVS-11, SAD B19, EBLV-1, and EBLV-2, cell culture supernatant from hybridoma clone 16DB4 was used (1:200), while the G protein of MOKV was detected with the polyclonal anti-MOKV-G immune serum (1:500) produced in rabbits. The cells were washed three times with PBS (4 °C) and then fixed for 30 min at room temperature with 3% paraformaldehyde (AppliChem, Darmstadt, Germany). The cells were washed twice with PBS containing 0.1 M glycine (AppliChem) and once with PBS and subsequently incubated with goat anti-mouse IgG AlexaFluor-488 conjugate (1:400, Life Technologies) and goat anti-rabbit IgG AlexaFluor4-88 conjugate (1:400, Life Technologies), each for 1 h at room temperature. Finally, the cells were washed twice with PBS and once with distilled water and embedded in Mowiol 4-88 (Sigma) mounting medium.

### 2.5. Cell Surface Biotinylation

Transgenic T-Rex™-CHO cell lines were seeded in 6-well culture plates (500,000 cells/well) and cultured for 24 h at 37 °C with either normal medium or medium containing 2 µg/mL doxycycline. The cells were washed three times with ice-cold PBS and incubated for 40 min on ice with 0.5 mg/mL of Sulfo-NHS-LC-Biotin (Life Technologies). The labeling reaction was stopped by washing the cells once with PBS containing 0.1 M glycine and incubating them in the same solution for 15 min at 4 °C. Subsequently, the cells were lysed in 1 mL of Nonidet P-40 lysis buffer (50 mM Tris/HCl, pH 7.5, 150 mM NaCl, 0.5% sodium deoxycholate (Sigma-Aldrich), 1% Nonidet P-40 (Sigma-Aldrich), protease inhibitor cocktail (Sigma-Aldrich, P8340). Thereafter, insoluble material was removed by centrifugation (20,000× *g*, 30 min, 4°C). Clarified cell lysate (10 µL) was mixed with 10 µL of 2× sodium dodecyl sulfate (SDS; Sigma-Aldrich) sample buffer containing 10% (v/v) 2-mercaptoethanol (Sigma-Aldrich), heated for 5 min at 90 °C, separated by SDS 10% polyacrylamide gel electrophoresis, and transferred to Porablot 0.45 µm pore size nitrocellulose membranes (Macherey-Nagel, Oensingen, Switzerland) by semidry blotting (0.8 mA/cm^2^, 60 min). The membrane was incubated overnight with blocking reagent (LI-COR Biosciences, Bad Homburg, Germany), washed three times with PBS containing 0.1% Tween 20 (AppliChem), and subsequently incubated for 60 min with IRDye^®^ 800CW streptavidin (1:2000; LI-COR Biosciences). The nitrocellulose membrane was washed three times with PBS/0.1% Tween 20 and once with detergent-free PBS. The biotinylated proteins were detected using the Odyssey Infrared Imaging System (LI-COR Biosciences).

### 2.6. Generation of Pseudotype Virus

VSV*∆G(FLuc), a G-deficient VSV-encoding green fluorescent protein (GFP) and firefly luciferase (FLuc), has been described previously [28]. For generation of a corresponding virus encoding secreted Nano luciferase (sNLuc), the sNLuc gene was amplified from the pNL1.3 plasmid (Promega, Dübendorf, Switzerland) and inserted into the XhoI and NheI endonuclease restriction sites of the pVSV*∆G(Luc) genomic plasmid thereby replacing the firefly luciferase gene. Recombinant VSV*∆G(sNLuc) was rescued on BHK-G43 helper cells following transfection of cDNA as described previously [28]. VSV*∆G(FLuc) and VSV*∆G(sNLuc) were propagated on transgenic T-Rex™-CHO cells in the presence of doxycycline (2 μg/mL). The *trans*-complemented particles were stored at −70 °C in the presence of 5% FBS. Pseudotype viruses were titrated on Vero cells in 96-well cell culture plates as described previously [28]. The virus titers were expressed as focus-forming units per mL (f.f.u./mL). Pseudotype viruses were passaged 3–4 times on helper cells before used in virus neutralization assays.

### 2.7. Virus Neutralization Tests

Unless otherwise indicated, immune sera were heated to 56 °C for 30 min in order to inactivate complement factors. The RABV neutralization activity of sera from vaccinated mice was determined using the modified RFFIT test as described previously [29,30]. For the pseudotype virus neutralization (PVN) test, two-fold serial dilutions of the immune sera or mAb 16DB4 were prepared in GMEM cell culture medium containing 5% FBS. The diluted sera were added to 96-well cell culture plates (50 µL/well, quadruplicates for each dilution) and incubated for 60 min at 37 °C with pseudotype virus (50 µL/well containing 100 f.f.u.). Vero cell suspension was added to the wells (100,000 cells/mL, 100 µL/well) and incubated at 37 °C for at least 8 h. Infection was monitored by fluorescence microscopy taking advantage of the GFP reporter protein. For each serum dilution, four wells were scored for the absence or presence of GFP-expressing cells (protected or non-protected wells) and neutralization doses 50% (ND_50_) were calculated according to the Spearman-Kärber method [31].

The PVN test was also performed with VSV*∆G(sNLuc) pseudotype viruses. Following incubation with serially diluted immune sera, the pseudotype virus/immune serum mixture (100 µL) was added to confluent Vero cells grown in 96-well microtiter plates and incubated for 60 min at 37 °C. The cells were washed twice with GMEM medium and subsequently incubated with 100 µL GMEM medium per well. At 6 h post-infection, 25 µL of cell culture medium was transferred to a white 96-well microtiter plate and Nano-Glo luciferase substrate (Promega) was added to each well (25 µL/well). Luminescence was recorded for 1 s with a Centro LB 960 luminometer (Berthold Technologies, Bad Wildbad, Germany). If the luminescence signal crossed the threshold value of 200 relative light units (RLU), the cell population in the well was regarded as infected, while below this value the cells were considered as negative. ND_50_ values were calculated according to Spearman and Kärber [31].

### 2.8. Statistical Analysis

Mean values and standard deviations were calculated. Data were analyzed by Mann-Whitney and Wilcoxon tests and *p* < 0.05 was considered significant.

## 3. Results

### 3.1. Generation of Transgenic Helper Cell Lines

**Figure 1:**
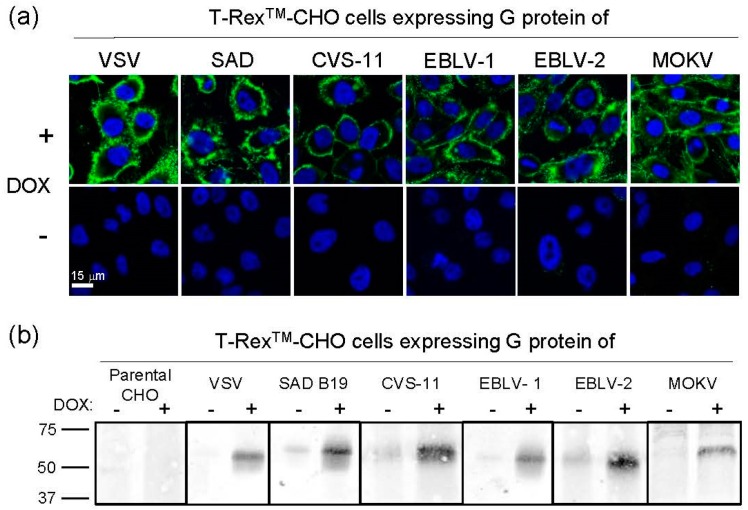
Conditional expression of rhabdoviral G proteins by transgenic helper cell lines. T-Rex™-Chinese Hamster Ovary (CHO) cells were stably transfected with recombinant pcDNA™ 4/TO plasmid encoding the indicated envelope G protein gene, selected with antibiotics, and cloned by limiting dilution. The cell clones were treated for 8 h with doxycycline (DOX) and inoculated with vesicular stomatitis virus (VSV)*ΔG. Clones that supported replication were selected and analyzed for expression of G protein: (**a**) Cell surface expression of the envelope G proteins was detected by indirect immunofluorescence using mAb I1 (VSV G protein), mAb 16DB4 (for detection of the G proteins of rabies virus (RABV), European bat lyssavirus type 1 (EBLV-1), and European bat lyssavirus type 2 (EBLV-2)), and rabbit anti-MOKV (Mokola virus) G protein serum as primary reagents; (**b**) Analysis of G protein expression by Western blot. Selected cell clones were treated with DOX for 8 h or left untreated, labeled with a membrane-impermeable biotinylation reagent, and lysed with NP-40 buffer. Solubilized cellular proteins were separated by sodium dodecyl sulfate-polyacrylamide gel electrophoresis (SDS-PAGE) under reducing conditions and blotted onto a nitrocellulose membrane. After incubating the membrane with a streptavidin-IRDye800 conjugate, the biotinylated cell surface proteins were detected with an Odyssey infrared scanner.

It has been previously shown that VSV lacking the glycoprotein G (VSVΔG) can be efficiently *trans*-complemented with the VSV G protein using a transgenic cell line [22]. This cell line was engineered to express the VSV G protein in a conditional manner in order to accommodate the cytotoxic properties of the protein [32]. Here, a similar approach was used for pseudotyping of VSVΔG with lyssavirus envelope glycoproteins. T-Rex™-CHO cells were transfected with the pcDNA™ 4/TO/Myc-His plasmid vector encoding the G protein of either VSV (serotype Indiana), rabies challenge virus standard 11 (CSV-11), rabies virus vaccine strain Street-Alabama-Dufferin B19 (SAD-B19), EBLV-1, EBLV-2, or MOKV (strain Ethiopia-16). The cells were selected with the antibiotics zeocin and blasticidin and subsequently cloned by limiting dilution. Following induction of transgene expression by doxycycline, cell clones were selected for their ability to support propagation of a recombinant G-deleted VSV expressing GFP (VSV*ΔG). The expression of the viral envelope glycoproteins by the selected cell clones was investigated by indirect immunofluorescence (Figure 1a). All G proteins could be detected only if the cells had been treated before with doxycycline. The antibodies reacted with intact, non-permeabilized cells indicating that the G proteins were expressed at the cell surface. Western blot analysis revealed that the G proteins had the expected molecular mass (Figure 1b). Using this approach, very little expression of G proteins was observed in non-induced cells.

### 3.2. Generation of VSV Pseudotypes

**Figure 2:**
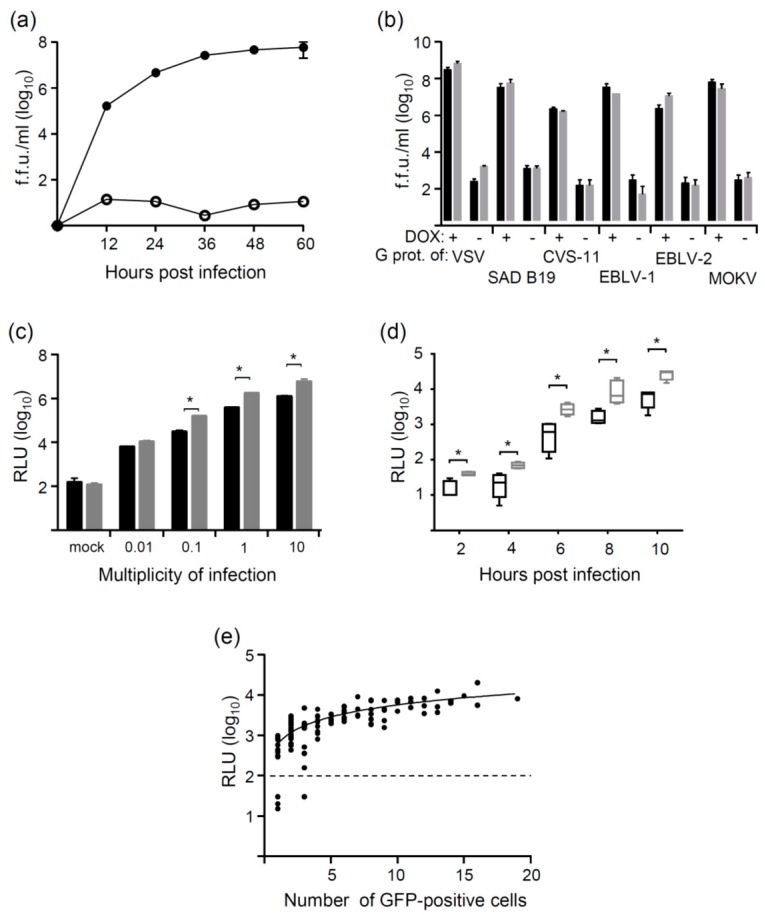
Conditional replication of VSV pseudotypes on helper cell lines. (**a**) The helper cell line expressing the G protein of Street-Alabama-Dufferin B19 (SAD B19) was treated for 8 h with doxycycline (DOX; filled symbols) or were left untreated (open symbols). Following infection of the cells with VSV*∆G(FLuc) (multiplicity of infection (m.o.i.) of 0.05), cell culture supernatants were collected at the indicated times and virus titers determined on Vero cells taking advantage of green fluorescent protein (GFP) reporter expression; (**b**) Pseudotype virus yield on induced and non-induced helper cells. Helper cell lines expressing the G proteins either of VSV, SAD B19, challenge virus standard 11 (CVS-11), EBLV-1, EBLV-2, or MOKV were treated for 8 h with DOX or were left untreated. The cells were infected VSV*∆G(FLuc) (dark bars) and VSV*∆G(sNLuc) (grey bars) using an m.o.i. of 0.05. Cell culture supernatants were collected at 36 h post infection (p.i.) and titrated on Vero cells; (**c**) Pseudotype viruses were produced on T-Rex™-CHO helper cells expressing the G protein of SAD B19. Vero cells were infected with either VSV*∆G(FLuc) (black bars) or VSV*∆G(sNLuc) (grey bars) using the indicated m.o.i. At 6 h p.i., luciferase activity was measured in cell lysates and cell culture supernatant, respectively; (**d**) Time kinetics of luciferase reporter expression. Vero cells were infected with SAD B19 G protein-pseudotyped VSV*∆G(FLuc) (black plots) or VSV*∆G(sNLuc) (grey plots) using an m.o.i. of 0.01. Luciferase activity was measured at the indicated times in cell lysates and cell culture supernatants, respectively; (**e**) Vero cells were grown in 96-well microtiter plates and infected with VSV*∆G(sNLuc) using m.o.i. ranging from 0.0001 to 0.001. The number of GFP-expressing cells was enumerated in 95 wells and plotted against the Nano luciferase activity that was secreted into the cell culture supernatant of the respective wells. The dashed line indicates the luminescence cutoff value above which the cells of a well were regarded as infected. f.f.u.: focus-forming units; RLU: relative light units. * *p* < 0.05.

For pseudotyping of VSV with rhabdoviral glycoproteins, either recombinant VSV*∆G(FLuc) expressing the two reporter genes GFP and FLuc [28] or VSV*∆G(sNLuc) expressing GFP and secreted sNLuc was used. In order to assess the efficacy of pseudotype particle production, the T-Rex™-CHO(SAD-G) helper cell line was infected with VSV*∆G(FLuc) using a multiplicity of infection (m.o.i.) of 0.05 infectious particles per cell. Aliquots of cell culture supernatants were collected at 12, 24, 36, 48, and 60 h post infection (p.i.) and infectious titers determined on Vero cells. Doxycycline-treated T-Rex™-CHO(SAD-G) cells produced maximum titers of about 10^8^ fluorescent focus-forming units/mL (f.f.u./mL) at 48 h p.i., whereas only low amounts of virus (<10^2^ f.f.u./mL) were released in the absence of doxycycline (Figure 2a). To determine the yield of pseudotype virus on different helper cell lines, the cells were infected with VSV*∆G(FLuc) or VSV*∆G(sNLuc) using an m.o.i. of 0.05. Cell culture supernatants were collected at 36 h p.i. and infectious virus titers determined on Vero cells. As expected, the highest virus yield (>10^8^ f.f.u./mL) was obtained with T-Rex™-CHO(VSV-G) cells (Figure 2b). The pseudotype virus titers achieved on helper cell lines expressing lyssavirus G proteins ranged from 10^7^ to 10^8^ f.f.u./mL. However, T-Rex™-CHO(CVS11-G) cells produced significantly lower pseudotype virus titers (about 10^6^ f.f.u./mL). This might be attributed to the pronounced neurotropism of CVS-11 and possibly lower expression levels of appropriate receptors in CHO cells. On all helper cell lines, infectious titers of VSV*∆G(FLuc) and VSV*∆G(sNLuc) did not differ (Figure 2b). Of note, significant levels of infectious pseudotype virus were only produced in the presence of doxycycline, indicating that the viruses were not able to propagate in the absence of G protein. Accordingly, non-helper cells (e.g., Vero cells) did not release any progeny virus following infection with any of the pseudotype viruses (data not shown).

Infectious pseudotype virus titers were determined on Vero cells taking advantage of GFP reporter protein expression. To elucidate how virus dose would correlate with luciferase reporter expression, Vero cells were infected with SAD G protein-pseudotyped VSV*∆G(FLuc) and VSV*∆G(sNLuc) using different m.o.i. As expected, the luminescence signals recorded 6 h p.i. were proportionally correlated with the virus dose used, i.e., a 10-fold higher m.o.i. resulted in an approximately 10-fold higher luminescence signal (Figure 2c). The secreted Nano luciferase activity produced higher signals than the firefly luciferase, suggesting that this enzyme represents a more sensitive reporter. To find out which infection time is minimally required to obtain a significantly high luciferase signal, Vero cells were infected with SAD G protein-pseudotyped VSV*∆G(FLuc) and VSV*∆G(sNLuc) using an m.o.i. of 0.01. Luminescence could be detected at significantly high levels as early as 6 h p.i. The sNLuc reporter always produced significantly higher signals than FLuc (Figure 2d).

In order to correlate the number of infectious pseudotype particles with luciferase reporter activity, Vero cells grown in 96-well microtiter plates were infected with VSV*∆G(sNLuc) using an m.o.i. ranging from 0.0001 to 0.001 infectious particles per cell. At 20 h p.i., the number of GFP-expressing cells was determined for 95 individual wells and correlated with the luciferase activity in the corresponding cell culture supernatants (Figure 2e). The mean luciferase activity per GFP-positive cell was calculated to be 572 ± 334 relative light units (RLU)/cell.

### 3.3. Neutralization of Pseudotype Virus

**Figure 3:**
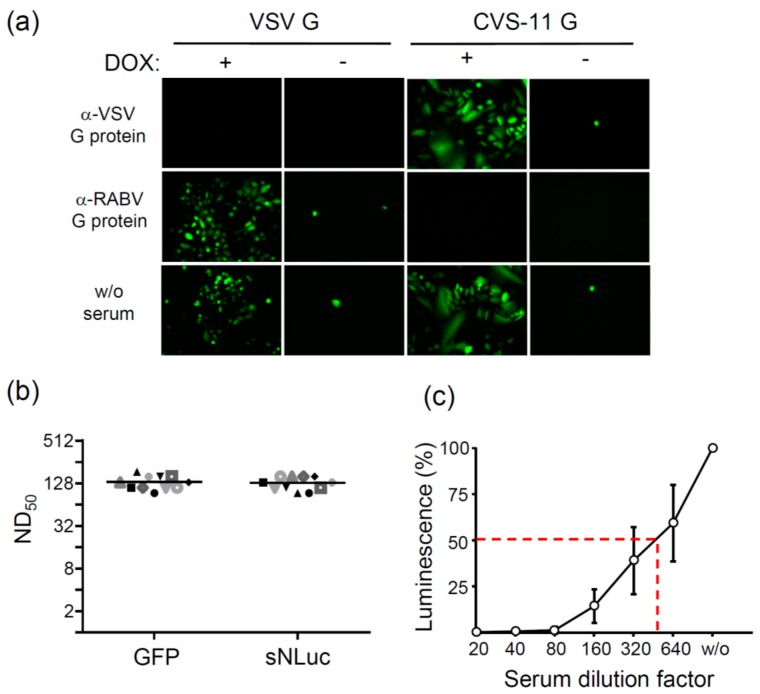
Pseudotype virus neutralization (PVN) test using the GFP reporter for detection. VSV*∆G(FLuc) was *trans*-complemented with G protein from either VSV or CVS-11. (**a**) The *trans*-complemented viruses (100 f.f.u.) were incubated for 60 min with either anti-RABV reference serum (2 IU/mL, 1:80), anti-VSV serum (1:100) or without (w/o) any serum and subsequently inoculated with either T-Rex™-CHO-SAD-G helper cells (if virus was *trans*-complemented with VSV G protein) or T-Rex™-CHO-VSV-G helper cells (if virus was *trans*-complemented with CVS-11 G protein) in the presence or absence of DOX. Expression of GFP was visualized 24 h post inoculation by fluorescence microscopy; (**b**) Comparison of the GFP and luciferase reporter read-outs. Serial two-fold dilutions of human anti-RABV reference serum (2 IU/mL) were incubated in quadruplicates for 60 min with 100 focus-forming units (f.f.u.) of VSV*∆G(sNLuc) that was *trans*-complemented with the CVS-11 G protein. Vero cells were added to the wells and incubated at 37 °C for 20 h. GFP expression was monitored by fluorescence microscopy. In parallel, the secreted Nano luciferase (sNLuc) activity was determined in the cell culture supernatant. The luciferase endpoint titers were calculated according to the method by Spearman–Kärber using a cutoff value of 200 RLU (see Figure 2e). The mean values of 11 independent experiments are shown; (**c**) The luciferase data were also used to calculate the serum dilution causing a reduction of luciferase by 50% (red dashed line). The luminescence recorded following infection of the cells in the absence of antibody was set to 100%. Mean values (*n* = 11) and standard deviations are shown.

In order to see whether CVS-11 G protein-pseudotyped VSV*∆G(Luc) would be sensitive to neutralization by immune sera directed to the RABV G protein, the virus (100 f.f.u.) was incubated for 60 min with either human anti-RABV serum, rabbit anti-VSV serum, or no serum at all. Subsequently, the pseudotype virus-serum mixtures were incubated with T-Rex™-CHO(VSV-G) or T-Rex™-CHO(SAD-G) helper cells, either in the presence or absence of doxycycline. At 18 h p.i., GFP expression was visualized by fluorescence microscopy (Figure 3a). In non-induced helper cells, GFP expression was restricted to single cells whereas in doxycycline-induced cells, virus was able to propagate, which led to an increased number of GFP-positive cells, thereby facilitating the detection of non-neutralized virus. Anti-RABV serum (1:80) completely neutralized CVS-11 G protein-pseudotyped VSV*∆G(FLuc) but had no effect if the virus had been *trans*-complemented with VSV G protein. Vice versa, anti-VSV immune serum (1:100) efficiently neutralized VSV G protein-pseudotyped but not CVS-11 G protein-pseudotyped VSV*∆G(FLuc), indicating that the *trans*-complementing envelope glycoprotein specifically determined the susceptibility of the virus to antibody-mediated neutralization.

To determine the neutralization dose 50% (ND_50_) of the WHO reference serum with the PVN test, 100 f.f.u. of CVS-11 G protein-pseudotyped VSV*∆G(sNLuc) were incubated with serial two-fold dilutions of reference serum and then inoculated with Vero cells. GFP reporter expression was monitored 8 h p.i., and wells completely devoid of GFP-positive cells were regarded as protected. The test was run in four parallels and ND_50_ values calculated according to the Spearman–Kärber method (Figure 3b). The PVN test was also performed using the sNLuc reporter and a luminescence cutoff value of 200 (see Figure 2e). If higher sNLuc activity was detected in the culture medium, the cells in this well were regarded as infected and the virus considered as not neutralized. Using this luminescence cutoff value, a mean ND_50_ value of 133 was calculated which corresponded well with the ND_50_ value of 134 which was obtained with the GFP read-out of the PVN test (Figure 3b). However, the serum dilution causing 50% inhibition of sNLuc reporter expression was calculated to be 1/500 (Figure 3c), suggesting that this read-out may offer a more sensitive way to detect neutralizing antibodies.

**Figure 4:**
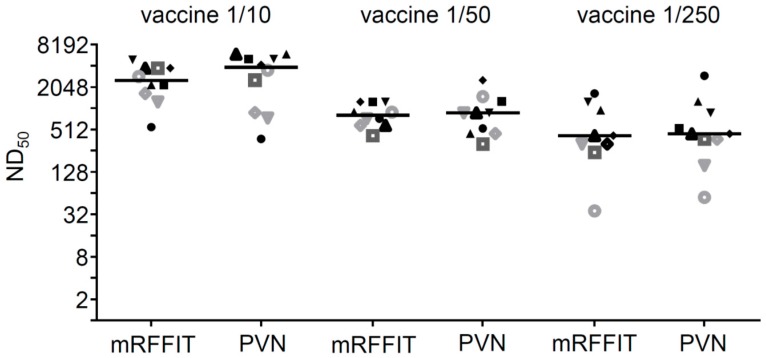
Comparison of the PVN test with the modified rapid fluorescent focus inhibition test (mRFFIT). Serum samples (not treated at 56 °C) from mice immunized with the indicated vaccine doses were analyzed with either the modified RFFIT or the PVN test. The PVN test was performed with CVS-11 G protein pseudotyped VSV*∆G(sNLuc) using the GFP reporter read-out. The neutralization dose 50% (ND_50_) values for individual animals (represented by different symbols) and the mean values for each vaccine group (*n* = 10) are shown.

In order to compare the PVN test with a commonly used RABV neutralization test, the assay was run with immune sera collected from mice that had been immunized with decreasing doses of RABV vaccine. The ND_50_ values of these sera were calculated and compared with the ND_50_ values determined by the modified RFFIT [29,30]. With both methods, the antibody responses of the immunized mice were found to depend on the vaccine dose with the highest ND_50_ values associated with the 1/10 vaccine group and the lowest with the 1/250 vaccine group (Figure 4). No significant differences were observed between the mean ND_50_ values calculated with either of the two methods.

### 3.4. Analysis of Pseudotype Viruses Bearing Other Lyssavirus Glycoproteins

Although infection with RABV is the most common cause of rabies disease, infection with other lyssaviruses has been associated with human fatalities as well [33]. Therefore, the PVN test was adapted to allow the detection of neutralizing antibodies directed to EBLV-1 and EBLV-2, which belong to phylogroup 1, and MOKV, which belongs to phylogroup 2. In addition, we compared CVS-11 G protein-pseudotyped with SAD G protein-pseudotyped VSV*∆G(FLuc) in order to see whether the antigenic properties of these G proteins differ. The neutralization of all these pseudotype viruses was tested with WHO reference serum (2 IU/mL), mAb 16DB4 (raised against the SAD vaccine strain), rabbit anti-CVS-11 G protein, and rabbit anti-MOKV G protein using the GFP reporter for detection of infected cells (Table 1). The WHO reference serum and mAb 16DB4 neutralized CVS-11 G protein- and SAD G protein-pseudotyped viruses with similar efficacy, whereas anti-CVS-11 serum neutralized CVS-11 G protein-pseudotyped virus significantly better than virus *trans*-complemented with the SAD G protein. The WHO reference serum, mAb 16DB4, and rabbit anti-CVS-11 serum also showed neutralizing activity against EBLV-1 or EBLV-2 G protein-pseudotyped VSV*∆G(Luc), however, the ND_50_ values were very low. Immune serum raised against MOKV showed neutralizing activity exclusively against MOKV G protein-pseudotyped virus but not against any of the other pseudotype viruses tested. Vice versa, none of the other immune sera tested was able to neutralize pseudotype virus containing the MOKV G protein. These results provide further evidence for the specificity of the PVN test and also demonstrate that the assay can be easily adopted to allow the detection of neutralizing antibodies directed to other lyssaviruses.

## 4. Discussion

The RFFIT and FAVN tests are currently the methods of choice for the detection and quantification of RABV neutralizing antibodies. Because these assays require the handling of live RABV, appropriate biosafety measures have to be conducted including vaccination of all laboratory workers and testing of their anti-RABV serum antibody levels on a regular basis. However, the RABV vaccine does not provide full protection against lyssaviruses other than RABV, in particular viruses of phylogroup 2 are not recognized by anti-RABV antibodies [15]. The RFFIT test is rather time-consuming because cells have to be incubated for 24 h prior to detection of infected cells by direct immunofluorescence [30]. Recombinant RABV expressing GFP may facilitate detection of infected cells [34,35,36], but still is a hazardous pathogen. Pseudotyping of G-deficient, non-replicative RABV is also feasible but suffers from slower reporter gene expression [37].

**Figure 5:**
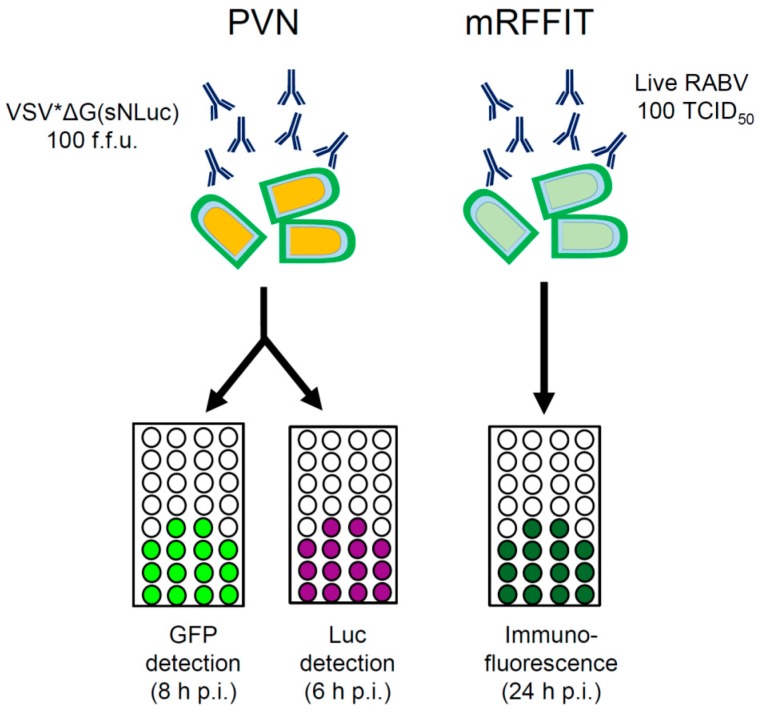
Performance of the PVN compared to the modified RFFIT. The PVN test makes use of propagation-incompetent pseudotype particles that express two reporter proteins, GFP and luciferase. The modified RFFIT makes use of live RABV and requires high biosafety standards. The different read-outs and time requirements of the assays are indicated. TCID_50_: median tissue culture infective dose.

In the present work, virus-neutralizing antibodies were detected taking advantage of propagation-incompetent pseudotype virus particles that comply with biosafety level 1 or 2 (depending on the country you are working in). The rapid and strong expression of VSV-encoded reporter genes (GFP, luciferase) facilitated the detection of virus-infected cells and allowed the assay to be performed within 8 h (Figure 5). In particular, the luciferase reporter turned out to be highly sensitive and allowed detection of even a single infected cell. The secreted Nano luciferase turned out to be superior over the firefly luciferase as it did not require prior lysis of the cells and produced stronger signals. Luminescence was recorded using a plate reader, which allowed the analysis of several samples in short time. The definition of a luciferase cutoff value enabled us to discriminate between positive and negative wells and to calculate ND_50_ values that were similar to those obtained with the GFP read-out (see Figure 3b). As an alternative read-out, the half maximal inhibitory serum/antibody concentration (IC_50_) leading to 50% reduction of luciferase reporter activity may be calculated. This would allow detection of even lower levels of neutralizing antibodies, however, the data cannot be directly compared with standard ND_50_ values. Compared to the conventional RFFIT and FAVN tests, which require a lot of counting of cells/foci with the risk of individual mistakes by the experimenter, the PVN test with the automated luciferase detection has the advantage of better reproducibility and provides the opportunity of better standardization.

The RFITT and FAVN tests require long incubation times in order to allow single non-neutralized virus to propagate. This is necessary to amplify the otherwise low immunofluorescence signal. A similar amplification can be achieved for the PVN test if doxycycline-induced helper cells are used for the assay rather than Vero or BHK-21 cells. On doxycycline-treated helper cells, the virus can perform two or more replication cycles, which will result in significant amplification of the reporter signal. Although the sensitivity of the PVN test is enhanced in this way, more time is needed to perform the assay. In order to avoid any interference of anti-RABV antibodies with the function of the *trans*-complementing envelope glycoprotein, it is recommended to use a helper cell line which expresses a heterologous glycoprotein, e.g., the VSV G protein.

Pseudotyping of VSV has been successfully performed with envelope glycoproteins derived from diverse viruses, indicating that incorporation of foreign viral glycoproteins into VSV particles occurs in a rather unspecific manner [38]. Nevertheless, VSV might be particularly well suited for the uptake and presentation of lyssavirus glycoproteins because VSV belongs to the same virus family and has the same bullet-shaped morphology as RABV. The envelope of both VSV and RABV is derived from the plasma membrane where budding takes place and contains a single-type trimeric glycoprotein of similar size. It is therefore likely that VSV pseudotype particles present lyssavirus glycoproteins in their proper conformation, similar or identical to the conformation presented by authentic RABV. This aspect is important since a considerable fraction of virus-neutralizing antibodies may bind to conformation-dependent epitopes [39,40].

Pseudotype particles can be produced quite easily by propagating VSV*ΔG(Luc) or VSV*ΔG(sNLuc) on helper cells expressing the viral glycoprotein of choice, e.g., RABV G protein. We used a conditional expression system because several viral glycoproteins, e.g., the VSV G protein [32], are known to exhibit cytotoxic properties which may interfere with any constitutive expression of these glycoproteins. The *trans*-complementation of VSV*ΔG by helper cells expressing the VSV G protein has never led to the emergence of propagation-competent VSV since this cell line was used for the first time more than 10 years ago [22]. Probably, the tight association of the VSV nucleocapsid protein with viral genomic RNA makes any recombination with mRNAs extremely unlikely. The *trans*-complementation of G-protein-deleted VSV may also be achieved by transient expression of viral glycoproteins. However, transfection of cells is laborious and expensive and often results in low and unsteady pseudotype virus titers. As an alternative to *trans*-complemented virus particles, chimeric VSV may be produced in which the VSV G gene is replaced by a foreign viral glycoprotein [38]. However, these propagation-competent viruses are free to mutate so that it cannot be ruled out that virulent viruses eventually evolve.

## 5. Conclusions

In conclusion, a safe, sensitive and reliable pseudotype virus neutralization test has been developed which allows rapid and sensitive quantification of antibodies with neutralizing activity against RABV and other lyssaviruses.

## Figures and Tables

**Table 1:** Neutralization of pseudotype viruses by immune sera.

	Neutralization Titer (ND_50_/mL) for VSV*∆G(Luc) Pseudotyped with the G protein of					
Antiserum/Antibody	VSV (Indiana)	RABV (SAD B18)	RABV (CVS-11)	EBLV-1	EBLV-2	MOKV (Eth-16)
α-VSV	1333	<4	<4	<4	<4	<4
α-RABV(RAI) ^a^	<4	191	224	19	38	<4
mAb 16DB4 ^b^	<4	13,881	8913	3452	5673	<4
α-CVS-11 G ^c^	<4	1608	5392	7	19	<4
α-MOKV G ^c^	<4	<4	<4	<4	<4	1831

^a^ Human anti-RABV WHO international immunoglobulin standard diluted to 2 IU/mL; ^b^ Mouse monoclonal antibody raised against the RABV SAD B19 vaccine strain; ^c^ Rabbit polyclonal antibodies directed against the G proteins of CVS-11 and MOKV. ND_50_: neutralization doses 50%; VSV: vesicular stomatitis virus; RABV: rabies virus; EBLV: European bat lyssavirus; MOKV: Mokola virus; mAb: monoclonal antibody; CVS: challenge virus standard.
